# The Roles of Amphibian (*Xenopus laevis*) Macrophages during Chronic Frog Virus 3 Infections

**DOI:** 10.3390/v13112299

**Published:** 2021-11-18

**Authors:** Muhammad Riadul Haque Hossainey, Amulya Yaparla, Kelsey A. Hauser, Tyler E. Moore, Leon Grayfer

**Affiliations:** Department of Biological Sciences, George Washington University, Washington, DC 20052, USA; riadulhaque@gwmail.gwu.edu (M.R.H.H.); amulya.yaparla@nih.gov (A.Y.); kahauser@gwmail.gwu.edu (K.A.H.); tmoore28@gwmail.gwu.edu (T.E.M.)

**Keywords:** amphibian, ranavirus, intestine, myeloid cells, interferons

## Abstract

Infections by Frog Virus 3 (FV3) and other ranavirus genus members are significantly contributing to global amphibian decline. The *Xenopus laevis* frog is an ideal research platform upon which to study the roles of distinct frog leukocyte populations during FV3 infections. Frog macrophages (MΦs) are integrally involved during FV3 infection, as they facilitate viral dissemination and persistence but also participate in immune defense against this pathogen. In turn, MΦ differentiation and functionality depend on the colony-stimulating factor-1 receptor (CSF-1R), which is ligated by CSF-1 and iterleukin-34 (IL-34) cytokines. Our past work indicated that *X. laevis* CSF-1 and IL-34 give rise to morphologically and functionally distinct frog MΦ subsets, and that these CSF-1- and IL-34-MΦs respectively confer susceptibility and antiviral resistance to FV3. Because FV3 targets the frog kidneys and establishes chronic infections therein, presently we examined the roles of the frog CSF-1- and IL-34-MΦs in seeding and maintaining these chronic kidney infections. Our findings indicate that the frog CSF-1-MΦs result in more prominent kidney FV3 infections, which develop into greater reservoirs of lingering FV3 marked by infiltrating leukocytes, fibrosis, and overall immunosuppressive states. Moreover, the antiviral effects of IL-34-MΦs are short-lived and are lost as FV3 infections progress.

## 1. Introduction

The diseases and population die-offs associated with amphibian infections by Frog Virus 3 (FV3) and other members of the genus Ranavirus (family *Iridoviridae*) are contributing to global amphibian population declines [1,2,3,4,5,6]. While the precise cell tropisms of these pathogens remain to be defined, macrophage (MΦ)-lineage immune cells are important to both the anti-ranaviral defenses as well as to the infection strategies of these pathogens [7,8]. While anuran (frogs/toads) amphibian hosts are able to clear the primary FV3 infections, residual virus persists within the frog kidneys and myeloid populations [9,10,11], presumably rendering the animals harboring this virus as reservoirs and sources of environmental dissemination of the pathogen. The transcriptional status of the persisting FV3 has not been well defined, with questions regarding whether this virus becomes quiescent or maintains an active transcriptional state during such prolonged/chronic infections.

It is not surprising that ranaviruses target MΦs, since the cells of this lineage are indispensable to host immunity and homeostasis. Indeed, although MΦs are important to recognizing and coordinating antiviral responses [12], MΦs are also commonly targeted by disparate viruses in establishing long-term viral reservoirs during chronic viral infections [13,14]. In turn, MΦ lineage-commitment, differentiation, survival, and functionality are controlled by the colony-stimulating factor-1 (CSF-1; M-CSF) receptor [15,16,17], which is ligated by CSF-1 and interleukin-34 (IL-34) cytokines/growth factors [18,19]. Using the *Xenopus laevis* frog model, we previously established that amphibian MΦs differentiated by IL-34 offer anti-FV3 protection, whereas CSF-1-MΦs render the animals significantly more susceptible to FV3 infections [7]. Our recent work indicates the functional dichotomy of these frog MΦ subsets stems at least in part from their distinct pathogen recognition capacities [20,21] and from more robust antiviral type I and III interferon cytokine production by the IL-34-MΦs [22]. Conversely, the mechanisms through which the frog CSF-1-MΦs compromise the animals to FV3 have remained largely unexplored.

Presently, we examine the roles played by the frog IL-34- and CSF-1-MΦs during chronic FV3 infections and establish the immunological parameters affected by skewing these frog MΦ populations during infections with this ranavirus pathogen.

## 2. Materials and Methods

### 2.1. Animals

*Xenopus laevis* outbred adult frogs (approximately six months to one-year old and around 1–1.5 inches in size) were purchased as tadpoles from Xenopus1 (Dexter, MI) and reared in-house. Animals were housed in an XR5 aquatic housing unit (IWAKI) with flow-through filtered (carbon and sediment filtration) and conditioned water at 21 °C. All infection studies were performed in, and all infected animals were housed in a designated quarantine/infection facility at 21 °C. Infected animals were housed in carbon- and sediment-filtered standing water in groups according to treatment. All animals were housed and handled under strict laboratory and IACUC regulations (Approval number 15-024).

### 2.2. Production of Recombinant CSF-1 and IL-34 Cytokines

*X. laevis* recombinant (r)CSF-1 and rIL-34 were produced as previously described [7]. Briefly, the CSF-1 and IL-34 sequences corresponding to the signal peptide-cleaved cytokines were ligated into the pMIB/V5 His A vector, and transfected into Sf9 insect cells (Cellfectin II, Invitrogen, Carlsbad, CA, USA). The positive transfectants were selected using 10 μg/mL blasticidin. Expression cultures were scaled up to 500 mL liquid cultures and grown for five days. The supernatants were collected by centrifugation and concentrated using polyethylene glycol flakes (8 kDa) at 4 °C followed by dialyzing overnight at 4 °C against 150 mM sodium phosphate. The recombinant cytokines in the concentrated supernatants were isolated using Ni-NTA agarose columns (Qiagen, Hilden, Germany) and washed with 2 × 10 volumes of high stringency wash buffer (0.5% tween 20, 20 mM sodium phosphate, 500 mM sodium chloride, 100 mM imidazole) and 5 × 10 volumes of low stringency wash buffer (all same, but with 40 mM imidazole). The rCSF-1 and rIL-34 were eluted in fractions with 250 mM imidazole and confirmed by western blot against the V5 tags on the proteins. The concentrations of recombinant proteins were determined using Bradford protein assays (BioRad, Hercules, CA, USA). A Halt protease inhibitor cocktail was added to rCSF-1 and rIL-34 and the recombinants were aliquoted and stored at −20 °C until use.

The recombinant control (r-ctrl) was generated by transfecting Sf9 cells with the empty pMIB/V5 His A expression vector (Invitrogen), grown and processed in parallel with rCSF-1 and rIL-34 production.

### 2.3. FV3 Stocks, Frog-Macrophage Enrichments, and Viral Infections

FV3 was generated following previously described methods [23]. Briefly, baby hamster kidney (BHK-21) cells were infected with FV3 at a multiplicity of infection of 0.1 and incubated at 30 °C and 5% CO_2_ for 5 days. The FV3-containing supernatants were pelleted through 30% sucrose by ultracentrifugation, re-suspended in saline, and viral titers were measured by plaque assays using BHK-21 cells.

MΦ enrichments and FV3 infections were performed as follows. Adult frogs (*n* = 15 for infection studies or *n* = 6 for gene expression studies) were intraperitoneally injected with 2.5 μg of rCSF-1 or rIL-34 in APBS or equal volumes of recombinant control (r-ctrl). After 3 days (optimal time for expanding and polarizing MΦs in vivo [7,24]), the frogs were intraperitoneally infected with 5 × 10^5^ plaque forming units per animal of FV3 (in APBS). Animal mortalities were monitored, frogs were sacrificed after 3-, 7-, 14-, 21-, or 28-days post infection (dpi), and their kidney tissues were collected for histology and DNA or RNA isolation.

### 2.4. Isolation of RNA and DNA from Kidney Tissues

For all experiments, kidney tissues were collected in Trizol reagent (Invitrogen) and stored at –80 °C until RNA and DNA isolation. Kidney tissues were homogenized by passage through progressively higher gauge needles and RNA was isolated according to the manufacturer’s instructions. DNA isolation was performed subsequent to RNA isolation. The DNA-containing layers were mixed with back extraction buffer (4 M guanidine thiocyanate, 50 mM sodium citrate, 1 M Tris pH 8.0), followed by centrifugation to collect DNA containing aqueous layer. The DNA was precipitated with isopropanol overnight at −20 °C. The next day, the DNA was pelleted by centrifugation, washed with 75% ice cold ethanol, and re-suspended in a TE (10 mM Tris pH 8.0, 1 mM EDTA) buffer. The DNA was then purified by phenol:chloroform extraction and re-suspended in molecular grade water. Both RNA and DNA concentrations were measured using NanoDrop (ThermoFisher, Waltham, MA, USA).

### 2.5. Quantitative Analysis of Gene Expression and FV3 Viral Load Assessment

Quantitative analysis of gene expression and FV3 DNA copy number has been previously described [24,25,26]. In brief, cDNA was synthesized from 500 ng of total RNA using iScript cDNA synthesis kits according to the manufacturers’ instructions (Bio-Rad, Hercules, CA, USA). Quantitative (q) RT-PCR was performed using 2.5 μL of cDNA as template. All the gene expression analyses were performed using the delta^delta CT method [27], examined relative to the *gapdh* endogenous control and normalized against the lowest observed expression.

Absolute qPCR analysis of FV3 DNA copy number was performed in comparison to serially diluted (10^1^–10^8^) FV3 vDNA Pol fragment-containing plasmid (pGEM-T vector; Promega, Madison, WI, USA), using 500 ng of isolated DNA from respective samples.

CFX96 Real-Time System and iTaq Universal SYBR Green Supermix were used in all the experiments and expression analyses were done using BioRad CFX Manager software (SDS). All primers used in this experiment were validated before use and all primer sequences are listed in Table S1.

### 2.6. Plaque Assay Analyses of FV3-Infected Frog Kidneys

Adult frogs were stimulated with rCSF-1, rIL-34 (2.5 μg/frog) or equal volumes of r-ctrl and three days later infected with FV3 (5 × 10^5^ PFU/frog; *n* = 5 per treatment group, per time point). After 7 and 21 days post infection (dpi), the animals were sacrificed, their kidneys were ascetically removed, homogenized in DMEM, serially-diluted and examined for FV3 loads by plaque assays, as described above.

### 2.7. Histological Analyses of Kidney Tissues

All histology was performed as described previously [28]. Briefly, adult kidney tissues were excised and immediately fixed in 10% neutral buffered formalin (VWR) and submitted to the GWU Pathology Core Lab for processing, paraffin embedding and sectioning. The paraffin-embedded tissue sections (5 μm) were stained with hematoxylin and eosin; or using Naphthol AS-D Chloroacetate (specific esterase, Leder; Sigma-Aldrich, St. Louis, MO, USA), or α-Naphthyl Acetate (non-specific esterase; Sigma-Aldrich) kits in accordance with the manufacturer’s instructions.

### 2.8. Statistical Analyses

Statistical analyses of the gene expression were conducted using one-way analyses of variance (ANOVA) with a post-hoc Tukey’s test. All statistical analysis was performed using R Statistical Software (version 2.14.0) or GraphPad Prism 7.0 software and *p* values < 0.05 were considered as statistically significant. The survival of FV3-infected animals was examined using a Kaplan-Meier procedure and Log-Rank (Mantel–Cox) test via GraphPad Prism 7.0 software, and found to be not statistically significant.

## 3. Results

### 3.1. CSF-1-MΦ-Enriched Frogs Succumb to FV3 Infections

**Figure 1:**
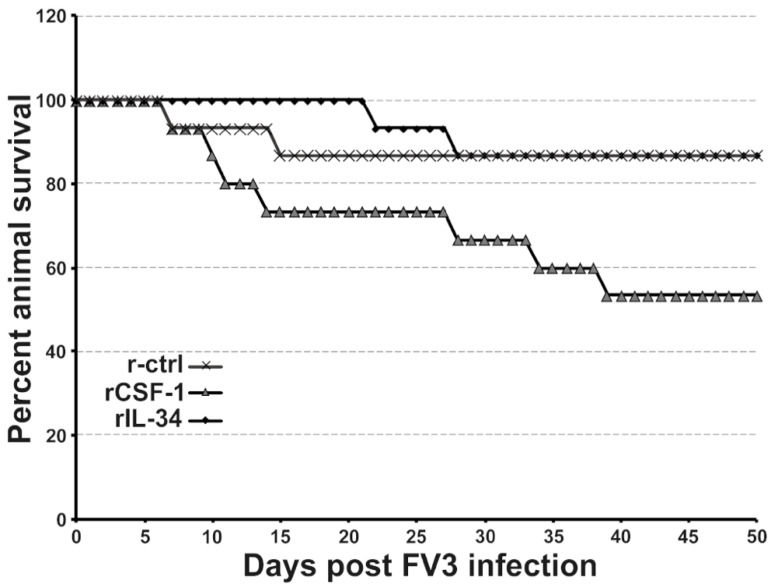
CSF-1-MΦ-administered frogs succumb to FV3 infections. *X. laevis* were injected ip with 2.5 μg of rCSF-1 or rIL-34 in APBS or equal volumes of a recombinant control (r-ctrl) and three days later infected ip with FV3 (5 × 10^5^ PFU). Animal survival was monitored over the course of 50 days, *n* = 15 per treatment group.

We previously established that *X. laevis* frogs may be enriched for CSF-1- or IL-34-MΦs by injecting these animals with recombinant (r)CSF-1 or rIL-34, respectively [29]. Whereas IL-34-MΦs offer anti-FV3 protection, CSF-1-MΦs render animals more susceptible to this pathogen [29]. Notably, while anuran (frogs and toads) tadpoles (including *X. laevis*) tend to succumb to FV3 infections, adult frogs generally clear FV3 infections without extensive mortalities [30,31,32,33,34]. Presently, we examined the roles of frog CSF-1- and IL-34-MΦs during long-term FV3 infection. To this end, we administered rCSF-1 or rIL-34 to frogs, and then challenged them with FV3. While our previous work indicated that enriching frog CSF-1-MΦs resulted in greater FV3 loads in these animals [7], we were surprised to find that expanding frog CSF-1-MΦs also led to an increase in FV3-infected animal mortalities, albeit not significantly so (Figure 1). Notably, in the days leading up to the respective animal mortalities, the succumbing frogs would begin to bloat and exhibited irregular swimming behavior. Necropsy of the succumbed animals indicated substantial hemorrhaging.

### 3.2. FV3 Persistence and Replication in CSF-1- and IL-34-MΦ-Enriched Frogs

**Figure 2:**
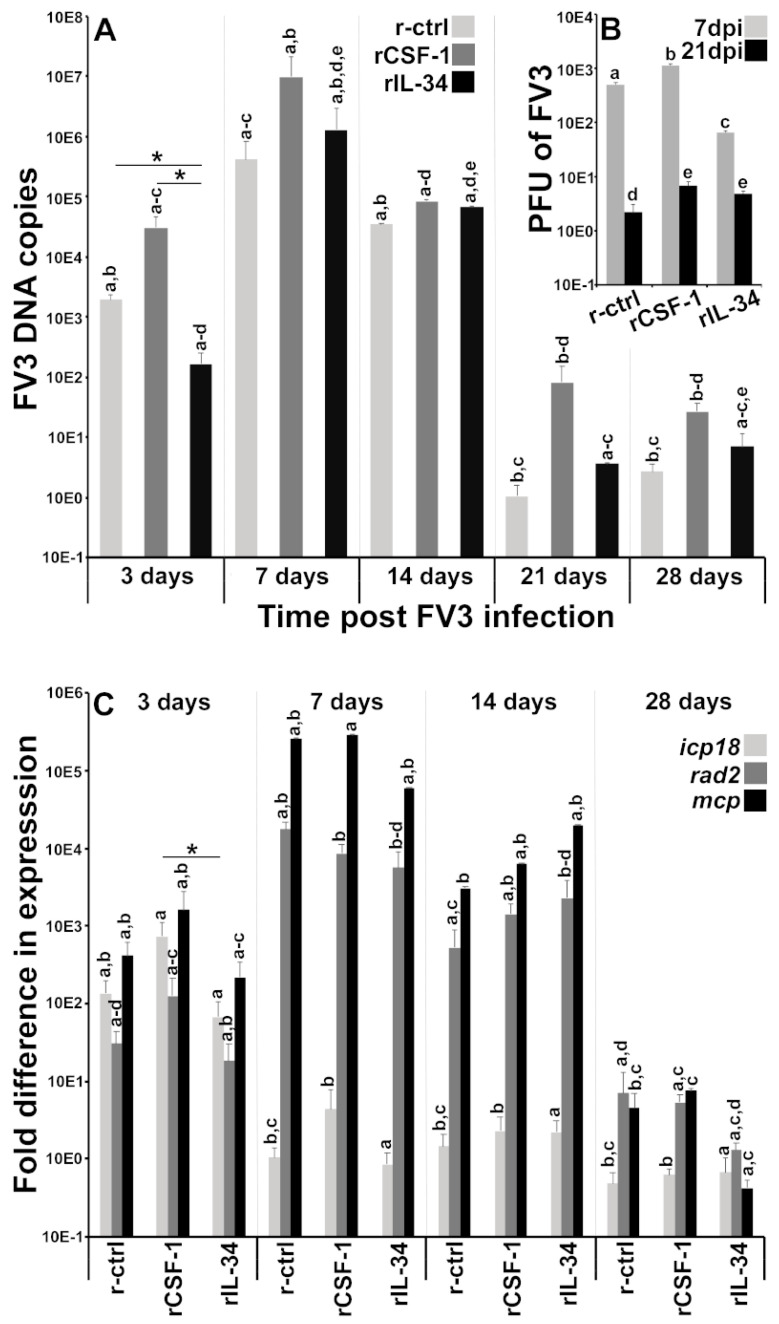
FV3 loads and gene expression analyses in control, CSF-1- and IL-34-MΦ-enriched frogs. *X. laevis* were injected ip with 2.5 μg of rCSF-1 or rIL-34 in APBS or equal volumes of empty vector control (r-ctrl) and three days later infected ip with FV3 (5 × 10^5^ PFU). At designated times, animals were sacrificed, and their kidneys were examined by qPCR for (**A**) FV3 DNA loads (*n* = 6), (**B**) by plaque assays for FV3 infectious viral particle content per kidney (*n* = 5) at 7 and 21 dpi, and (**C**) by qPCR for FV3 gene expression of *icp18*, *rad2* and *mcp* genes (*n* = 6). The results are means ± SE. The letters above head bars indicate statistical groups, with each letter representing those treatment groups that are not statically different from each other and distinct letters indicating treatment groups that are significantly different. Asterisks above lines (*) denote statistical differences between the treatment groups denoted by the lines, *p* < 0.05.

CSF-1- and IL-34-MΦ enrichments affect a number of frog tissues, chiefly amongst them the frog kidneys [7,22,24], which are a central target for FV3 replication [23]. Accordingly, we next examined the kidneys of MΦ-enriched, FV3-infected animals to discern how these shifts in MΦ populations affect short- and long-term infection outcomes. As expected, IL-34-MΦ-enriched frogs possessed significantly lower FV3 DNA loads in their kidneys after three days post-infection (dpi), but this protection was lost at later infection times (Figure 2A). Conversely, CSF-1-MΦ-enriched frogs harbored higher viral loads across all examined time points and as compared to recombinant control (r-ctrl; sups from empty expression vector-transfected insect cells, processed in parallel to rCSF-1/rIL-34)-injected and IL-34-MΦ-enriched frogs (Figure 2A). Akin to previous studies that established that residual FV3 persists withing infected frog kidneys after the immune clearance of the primary infections [10,11], we also detected persisting FV3 DNA in the kidneys of frogs from all treatment groups, albeit in substantially lower levels at 21 and 28 dpi (Figure 2B).

Our plaque assay analysis of MΦ-enriched, FV3-infected frog kidneys supported the above findings. Notably, at seven dpi, CSF-1-MΦ-enriched animals possessed significantly greater infectious FV3 particles than the r-ctrl-injected animals, whereas the IL-34-MΦ-enriched frogs had significantly lower FV3 loads in their kidneys (Figure 2B). After 21 dpi, animals possessed significantly decreased, although persisting FV3 infectious particles in their kidneys, with CSF-1- and IL-34-injected animals harboring significantly more viral particles in their kidneys than the r-ctrl-injected animals (Figure 2B).

In general, the above observations regarding differences in kidney viral DNA copies and infectious FV3 particles were also reflected in the viral gene expression (Figure 2C). The kidneys of IL-34-MΦ-enriched frogs exhibited lower transcript levels of the FV3 immediate early (*icp18*), delayed early (*rad2*) and late (*mcp*) genes than the kidneys of CSF-1-MΦ-enriched frogs at three dpi and the loss of this protection with infection time (Figure 2C). Likewise, at 28 dpi, we observed substantial, albeit reduced expression of all examined FV3 genes (Figure 2C). While the kidneys of CSF-1-MΦ-enriched frogs possessed greater FV3 DNA loads at 28 dpi (Figure 2A), we did not see difference in viral gene expression between the kidneys of control, CSF-1-MΦ- or IL-34-MΦ-enriched animals (Figure 2C).

### 3.3. CSF-1-MΦ-Mediated Susceptibility to FV3 Is Marked by Kidney Fibrosis and Leukocyte Infiltration

**Figure 3:**
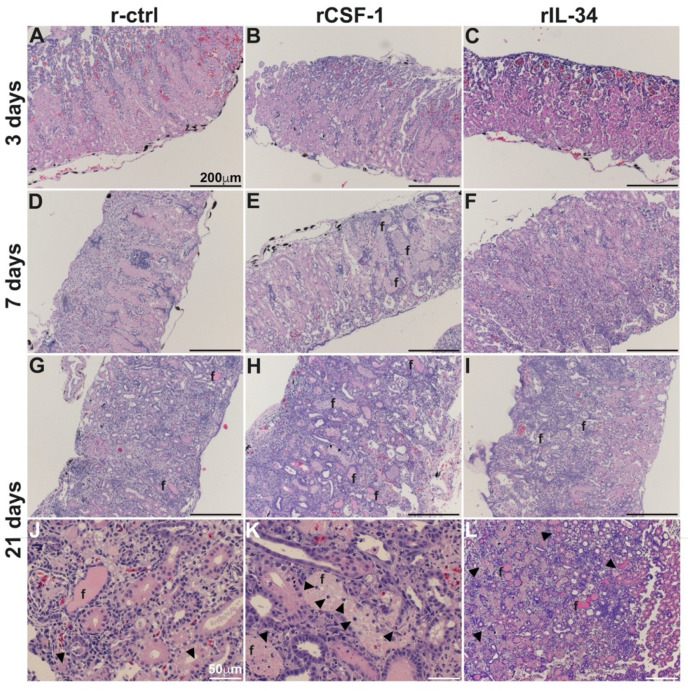
CSF-1-MΦ-enriched, FV3 infected frogs possess greater kidney tissue damage and infiltrating leukocytes. *X. laevis* were injected ip with 2.5 μg of rCSF-1 (**B**,**E**,**H**,**K**) or rIL-34 (**C**,**F**,**I**,**L**) in APBS or equal volumes of a recombinant control (r-ctrl; (**A**,**D**,**G**,**J**)) and three days later infected ip with FV3 (5 × 10^5^ PFU). At designated times, animals were sacrificed, and their kidneys were processed for histology and stained with hematoxylin & eosin and examined by microscopy. Fibrosis (f) and leukocytes (arrows) are indicated, and the images are representative of sections from four mock-infected and five infected animals per treatment group (*n* = 4 for uninfected controls and *n* = 5 for FV3-infected groups) individual animal kidneys from respective treatment groups and times.

To account for the greater mortality of FV3-infected frogs that had been enriched for CSF-1-MΦs and reconcile the greater FV3 loads in these animals, we next performed histological analyses of kidneys from r-ctrl-, rCSF-1- and rIL-34-injected and FV3 infected frogs after 3, 7 and 21 days of infection (Figure 3). At 3 dpi, we did not observe marked difference between the infected frog kidneys (Figure 3A–C), but by 7 dpi, the kidneys of CSF-1-MΦ-enriched animals possessed extensive fibrosis and tissue damage (Figure 3E) whereas the kidneys of control and IL-34-MΦ-enriched animals did not (Figure 3D,F). At 21 dpi, extensive fibrosis, tissue damage and loss of kidney architectures were observed in all treatment groups (Figure 3G–L), however the CSF-1-MΦ-enriched frog kidneys exhibited substantially greater tissue damage, accompanied by the infiltration of leukocytes (Figure 3H,K).

### 3.4. CSF-1-MΦ-Enriched Frogs Exhibit Greater Kidney Expression of Chemokine Genes

**Figure 4:**
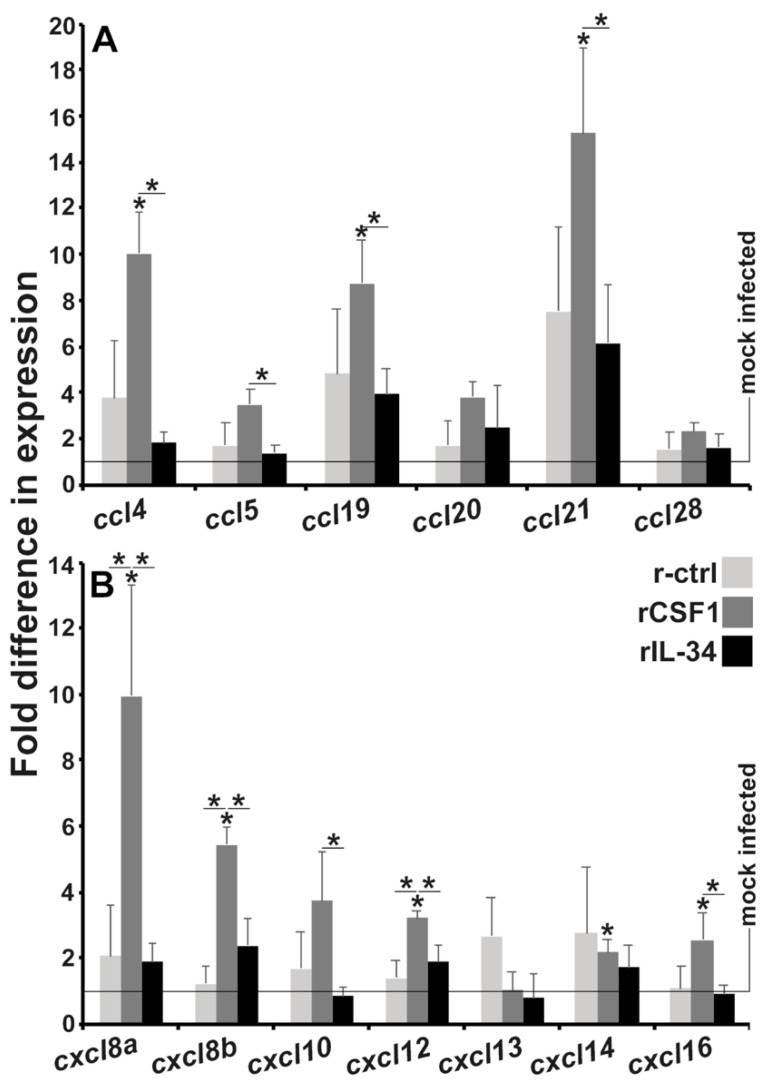
Kidneys from CSF-1-MΦ-enriched, and chronically FV3-infected frogs possess greater expression of chemokine genes. *X. laevis* were injected ip with 2.5 μg of rCSF-1 or rIL-34 in APBS or equal volumes of empty vector control (r-ctrl) and three days later infected ip with FV3 (5 × 10^5^ PFU) or mock-infected with ip APBS injections. After 28 days of infection, animals were sacrificed, and their kidneys were examined by qPCR for expression of (**A**) CC and (**B**) CXC motif chemokine genes. The results are means ± SE (*n* = 6). All expression was normalized against mock-infected controls, with average baseline (uninfected) expression indicated by horizontal lines. Asterisks (*) indicate significant increase in gene expression above mock-infected controls and asterisks above lines (*) denote statistical differences between the treatment groups denoted by the lines, *p* < 0.05.

Because we observed extensive leukocyte infiltration into the kidneys of CSF-1-MΦ-enriched, FV3-infected frogs at later infection times (Figure 3), we next examined the kidneys of r-ctrl, rCSF-1- and rIL-34-administered animals after 28 dpi for expression of a panel of CC and CXC chemokine genes (Figure 4A,B). As expected, the kidneys of CSF-1-MΦ-enriched, FV3-infected frogs exhibited greater expression of a number of these genes including *ccl4*, *ccl5*, *ccl19* and *ccl21* (Figure 4A) as well as *cxcl8a*, *cxcl8b*, *cxcl10*, *cxcl12* and *cxcl16* (Figure 4B), compared to the kidneys of control or IL-34-MΦ-enriched animals.

### 3.5. CSF-1-MΦ-Enriched Frogs Possess Greater Kidney Infiltration by Granulocytes and Macrophages

**Figure 5:**
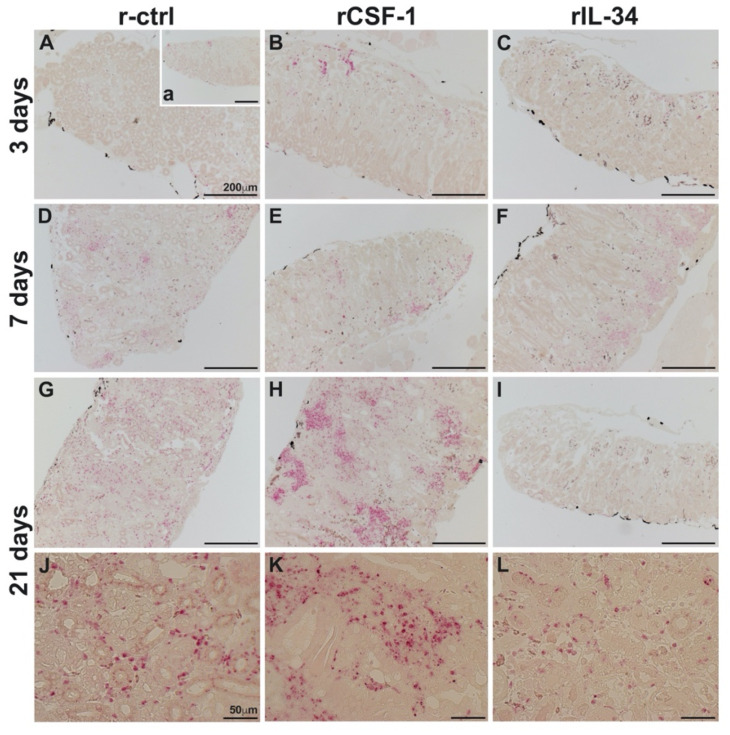
The kidneys of CSF-1-MΦ-enriched, FV3 infected frogs possess greater infiltration of granulocytes. *X. laevis* were injected ip with 2.5 μg of rCSF-1 (**B**,**E**,**H**,**K**) or rIL-34 (**C**,**F**,**I**,**L**) in APBS or equal volumes of a recombinant vector control (r-ctrl; (**A**,**D**,**G**,**J**); inset **a** panel: uninfected) and three days later infected ip with FV3 (5 × 10^5^ PFU). At designated times, animals were sacrificed, and their kidneys were processed for histology and examined by NASDCl-specific esterase (Leder) stain, with granulocytes staining pink. The images are representative of sections from kidneys of four mock-infected and five infected animals per treatment group (*n* = 4 for uninfected controls and *n* = 5 for FV3-infected groups). Inset panel in (**A**), denoted (a) is representative of kidneys from mock-infected animals.

**Figure 6:**
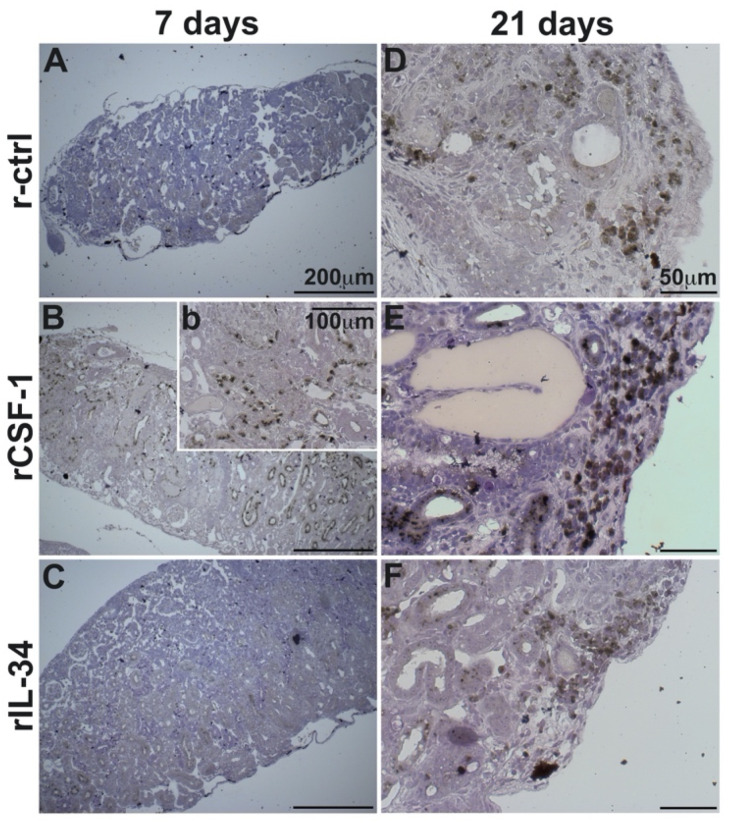
The kidneys of CSF-1-MΦ-enriched, FV3 infected frogs possess greater infiltration of MΦs. *X. laevis* were injected ip with 2.5 μg of rCSF-1 (**B**, inset b, **E**) or rIL-34 (**C**,**F**) in APBS or equal volumes of a recombinant control (r-ctrl; **A**,**D**) and three days later infected ip with FV3 (5 × 10^5^ PFU). At designated times, animals were sacrificed, and their kidneys were processed for histology and examined by α-Naphthyl Acetate (non-specific esterase; Sigma-Aldrich) stain, with MΦ-lineage cells staining brownish-black. The images are representative of sections from kidneys of five infected animals per treatment group (*n* = 5 for FV3-infected groups). Inset panel in (**B**), denoted as (b) is higher magnification of part of the same section.

Infection-associated tissue damage, such as that seen in the CSF-1-MΦ-enriched and FV3-infected frogs, is often accompanied by and is attributed to infiltrating inflammatory cells such as granulocytes and MΦs [35]. Moreover, many of the chemokines upregulated in these animals target such myeloid cells [36]. Accordingly, we next examined the kidneys of r-ctrl-, rCSF-1- and rIL-34-administered and FV3-challenged animals for infiltrating granulocytes and macrophages by specific and non-specific esterase stains (Figure 5 and Figure 6, respectively).

Strikingly, while the kidneys of FV3-infected control and the IL-34-MΦ-enriched frogs did not possess substantial granulocyte infiltration after three dpi (Figure 5A,C, respectively), the CSF-1-MΦ-enriched animals already showed substantial granulocyte infiltration (specific esterase positive cells) at this early time point (Figure 5B). By seven dpi, all experimental groups possessed comparable granulocyte infiltration of their kidneys (Figure 5D–F) but by 21 dpi, the CSF-1-MΦ-enriched animals had drastically more pronounced granulocytes within their kidney tissues (Figure 5H,K) than seen in the control (Figure 5G,J) or IL-34-MΦ-enriched (Figure 5I,L) animals at this time.

While we did not observe substantial macrophage infiltration into the kidneys of any treatment groups at three dpi, by seven dpi the kidneys of the CSF-1-MΦ-enriched frogs possessed substantial infiltration by macrophages (non-specific esterase; Figure 6Bb). By contrast, the control- and the rIL-34-administered, FV3-infected frogs lacked detectable macrophage infiltration into their kidneys at seven dpi (Figure 6A,C). By 21 dpi, all experimental groups possessed detectable macrophage accumulation in their kidneys, with the CSF-1-MΦ-enriched frogs displayed drastically more pronounced kidney infiltration by macrophages (Figure 6D–F).

### 3.6. Kidneys of CSF-1-MΦ-Enriched Frogs Possess Greater Expression of Genes Associated with Immune Suppression

**Figure 7:**
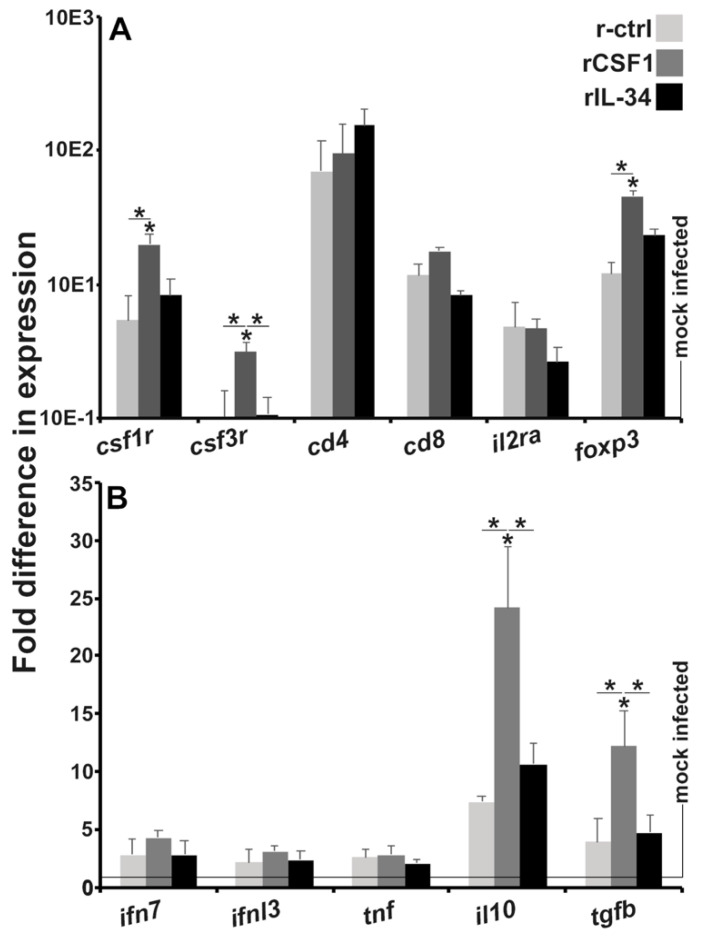
Kidneys from CSF-1-MΦ-enriched, and chronically FV3-infected frogs possess greater expression of myeloid cell markers and immunosuppressive genes. *X. laevis* were injected ip with 2.5 μg of rCSF-1 or rIL-34 in APBS or equal volumes of empty vector control (r-ctrl) and three days later infected ip with FV3 (5 × 10^5^ PFU) or mock-infected with ip APBS injections. After 28 days of infection, animals were sacrificed, and their kidneys were examined by qPCR for gene expression of (**A**) leukocyte markers and (**B**) cytokines. The results are means ± SE (*n* = 6). All expression was normalized against mock-infected controls, with average baseline (uninfected) expression indicated by horizontal lines. Asterisks (*) indicate significant increase in gene expression above mock-infected controls and asterisks above lines (*) denote statistical differences between the treatment groups denoted by the lines, *p* < 0.05.

To further corroborate the CSF-1-MΦ-enriched, FV3-infected frog kidney leukocyte infiltration, we examined the kidneys of r-ctrl-, rCSF-1- and rIL-34-administered frogs for expression of several hallmark leukocyte marker genes after 28 dpi (Figure 7A). As expected and in congruence with our chemokine gene expression (Figure 4) and histology data (Figure 5 and Figure 6), the kidneys of CSF-1-MΦ-enriched frogs possessed significantly greater expression of the frog granulocyte and macrophage markers [21], *csf1r* and *csf3r,* respectively (Figure 7A). All treatment groups had comparable kidney expression levels of *cd4* and *cd8* T cell co-receptor genes as well as similar expression of the high affinity interleukin-2 (*il-2*; T cell growth factor) receptor gene (Figure 7A). Interestingly, the kidneys of CSF-1-MΦ-enriched frogs also possessed significantly elevated expression of *foxp3* (Figure 7A), a marker of immunosuppressive T regulatory cells [37].

Following up on the elevated *foxp3* expression in the CSF-1-MΦ-enriched frog kidneys, we next examined the kidneys of the three treatment groups 28 dpi for the expression of antiviral (*ifn7*, *ifnl3*), proinflammatory (*tnf*) and immunosuppressive (*il10*, *tgfb*) cytokine genes (Figure 7B) to discern the overall immune status of these tissues. While all frogs possessed similar kidney mRNA levels of the antiviral and inflammatory cytokine genes, the kidneys of CSF-1-MΦ-enriched frogs exhibited significantly increased expression of the immunosuppressive (*il10*, *tgfb*) cytokine genes (Figure 7B).

## 4. Discussion

MΦ-lineage cells are believed to be key viral reservoirs during chronic FV3 infections [10]. Our past work indicated that during acute infections, the IL-34- and CSF-1-MΦs, respectively, control and facilitate these FV3 infections. In turn, our present work indicates that while the IL-34-MΦ-mediated anti-FV3 protection is short-lived, the increased frog susceptibility conferred by CSF-1-MΦs extends into chronic FV3 infections, exacerbating the infection outcomes. Moreover, our findings confirm past observations [10] that rather than becoming transcriptionally quiescent, the non-cleared proportion of FV3 maintain transcriptionally active chronic infections in the frog kidneys, including the presence of infectious virus particles therein. Moreover, several studies indicate that chronic FV3 infections are further exacerbated by inflammatory stimuli such as heat-killed *E. coli* [10] or TLR5 agonists [11]. It is possible that these inflammatory signals result in the mobilization and/or shifts in FV3-laden frog MΦs away from antiviral states (akin to IL-34-MΦs), thereby resulting in dissemination of the virus and greater replication within the reservoir MΦs (akin to CSF-1-MΦs) and the newly infected cells.

While our past studies indicated that both *il34* and *csf1* gene expression is increased in the kidneys of FV3-infected animals [24], it is difficult to speculate what proportion of the frog kidney MΦs adopt the IL-34- or CSF-1-like states. IL-34 mediates its biological roles as a soluble glycoprotein whereas the mammalian, and presumably the frog CSF-1 functions as a membrane bound protein (the frog CSF-1 possesses a transmembrane domain and several glycosylation sites), a secreted glycoprotein, and/or a secreted proteoglycan that interacts with extracellular matrices [38]. As such, it is likely that there are microenvironments within healthy, as well as infected frog kidneys (amongst other tissues) that are predominated by either IL-34- or CSF-1-MΦs, depending on the predominating cytokine type, form, and tissue microenvironment therein. Moreover, we previously showed that both the frog CSF-1- and IL-34-MΦs may be counter-polarized by the reciprocal stimulation with IL-34 and CSF-1 cytokines, respectively [22,39] and as such, it is likely that any ratios of CSF-1-/IL-34-MΦs residing within frog tissues are transient are subject to change with changing microenvironments. It this respect, it is notable that at seven dpi, rCSF-1- and rIL-34-injected frogs, respectively, possessed significantly greater and fewer FV3 loads than control animals. Conversely, by 21 dpi both the CSF-1- and IL-34-MΦ-enriched frogs possessed significantly greater FV3 infectious particles than the control frogs. Presumably, while the rIL-34-mediated MΦ polarization of frogs was short-lived, the MΦ-enrichment of these animals by this cytokine persisted throughout the course of the study. This enrichment would thus provide further means for FV3 persistence and dissemination, possibly due to these rIL-34-enriched cells also adopting a CSF-1-MΦ-like phenotype with infection time. It will be interesting to explore these notions further in the context of progressing FV3 infections and examine whether this virus manipulates the frog host tissue ratios away from the IL-34-MΦs towards the CSF-1-MΦs within and beyond the timeframe of the FV3 infection studies presented here.

Most amphibian chemokines have not been functionally characterized but are generally believed to facilitate the biological roles attributed to their respective mammalian counterparts. Many of these have been associated with pathology during certain chronic viral infections of mammals (reviewed in reference [40]). Chiefly amongst these, CCL3 (macrophage inflammatory protein-1α; MIP-1α), CCL4, CCL5, CXCL8, and CXCL10 are broadly expressed in the context of several (murine/human) viral infections [40]. Notably, we observed significantly higher expression of the frog counterpart chemokine genes encoding CCL4, CCL5, CXCL8 (CXCL8a and CXCL8b), and CXCL10 in the kidneys CSF-1-MΦ-enriched, FV3-challenged frogs, concurrent with greater pathology and leukocyte infiltration of this organ. The lack of available reagents precludes us for determining whether the CSF-1-MΦs are directly responsible for this elevated chemokine expression, and/or if these cells are resulting in elevated leukocyte recruitment by bystander kidney cells. Perhaps future frog FV3 infection studies in which the proportions of CSF-1- and IL-34-MΦs are altered, may shed additional light upon the dynamics and consequences of the recruitment of distinct leukocyte subsets into virally infected frog tissues.

CCL19 and CCL21, which were also upregulated in chronically infected CSF-1-MΦ-enriched frogs, are particularly known for homing of T cells and dendritic cells to lymphoid tissues, but also perform several other functions including regulatory and memory T cell function [41]. It is possible that the increased expression of these chemokines reflects the infiltration of the infected kidney tissues by lymphocytes and in particular regulatory T cells. Similarly, CXCL12 expression has been linked with immunosuppressed microenvironments [42], such as that seen in the kidneys of the CSF-1-MΦ-enriched, FV3 infected frogs. Conversely, chemokines such as CXCL8a and CXCL10, respectively, recruit proinflammatory granulocytes [43] and T cells [44], although we showed that CXCL8b is responsible for recruiting immunosuppressive granulocytes [28,45] while CXCL10 has also been linked with a number of chronic diseases [46]. Greater insights into the functional roles of these and other amphibian chemokines will shed more light into the involvement of specific leukocyte lineages and their activation states during infections with pathogens such as FV3.

We observed extensive infiltration of both granulocytes and MΦs into the kidneys of CSF-1-MΦ-enriched, FV3 infected frogs, which coincided with more extensive pathology therein, as compared to the kidneys of control- and IL-34-MΦ-enriched animals. This is consistent with what has been observed across other vertebrates, where both granulocyte- and MΦ-lineage cells may invade infected tissues, exacerbating tissue damage [47]. Notably, subsets of frog granulocytes [28,45] and certain populations of MΦ-lineage cells [48] are also integral to immune suppression and resolution of inflammatory responses. We suspect that our observations reflect dysregulated immune responses in the CSF-1-MΦ-enriched animals. It is possible that more leukocytes infiltrate the kidneys of these animals in an attempt to clear the greater FV3 loads and are retained therein due to the inability to eliminate these viral infections. Immune dysregulation, some form of tolerance and/or viral immune evasion may then account for the establishment of the immunosuppressive states seen in the kidneys of these CSF-1-MΦ-enriched frogs at later infection times, with the retained and/or newly recruited leukocytes contributing to this suppressive state.

Many elements of these studies are specific to amphibian innate and antiviral immunity and provide insight into the roles of frog MΦ subsets during chronic ranavirus infections. It is, however, notable that the dichotomy of CSF-1- and IL-34-MΦ functionality appears to be evolutionarily conserved [39,48]. As such, greater understanding of the roles of these MΦ subsets during chronic infections may grant new perspectives into not only amphibian but also into human diseases.

## Supplementary Materials

The following are available online at https://www.mdpi.com/article/10.3390/v13112299/s1, Table S1: List of primer sequences.
