# Supplementary material for: The Roles of Amphibian (Xenopus laevis) Macrophages during Chronic Frog Virus 3 Infections
Source: Viruses. 2021 Nov 18;13(11):2299. doi: 10.3390/v13112299 (PMC8621048; doi:10.3390/v13112299)
Supplement: Supplementary file 1 [file viruses-13-02299-s001.zip › viruses-1330871-supplementary.pdf]

Table S1. List of primer sequences

Primer	Sequence 5'→3'	Primer	Sequence 5'→3'
ccl4 F<br>*ccl4 R*	TCCAACCCTGGCGTTATATTC<br>GCATTCTTGTCCAGCTTTGTC	*cxcl12* F<br>*cxcl12* R	ATAAAAAACACATTCGTTCCT<br>ACAGCGGTTAACCACTTGG
ccl5 F<br>*ccl5* R	GTTCTTGTCACCCGGAAGAA<br>TGTGTTGCTTCAGGCATCT	*cxcl13* F<br>*cxcl13* R	AAGCTGACACGGGTTGAA<br>CAGCTTGTGGATTGACACATAC
ccl19 F<br>*ccl19* R	ACCATCAACAAGCCGATTCC<br>GAGCTGTATGTTTCTCCGAGTG	*cxcl14* F<br>*cxcl14* R	GGACTTGTACTGCCGTTCTT<br>GCGTACCTTCCCATGTAATGA
ccl20 F<br>*ccl20* R	CTCCTGGCTGCTTTGATGT<br>GGATGAAGAATACGGTCTGTGTAT	*cxcl16* F<br>*cxcl16* R	GTGGGTCCAGGAACTGATATG<br>GCTCAGAGCCCTTGGAATAA
ccl21 F<br>*ccl21* R	ATCCAGTGGCTGCTCAATC<br>CTGGCAATCATAGTCTGTACCC	*DNApol* F<br>*DNApol* R	CAAGAACGTGTGCTACTCCA<br>AGCCTCTCGTACTCTACCTTC
ccl28 F<br>*ccl28* R	GCTCTCTGTGTCAATCCCAATA<br>CCGGTGTTTCTTTCTTCCCT	*foxp3* F<br>*foxp3* R	ATGGCACGGTTGTCTGGAGA<br>CAAGCTGTTCTTCTAGTTTGTG
ccl20 F<br>*ccl20* R	CTCCTGGCTGCTTTGATGT<br>GGATGAAGAATACGGTCTGTGTAT	*gapdh* F<br>*gapdh* R	ATGTGTCCGTTGTGGATCTG<br>GATTCCTTTCATTGGTCCCTCT
ccl21 F<br>*ccl21* R	ATCCAGTGGCTGCTCAATC<br>CTGGCAATCATAGTCTGTACCC	*icp18* F<br>*icp18* R	TGTGTCTGGAGAACCCTACA<br>TGGTGATCTTGACTTGAAACTC
ccl28 F<br>*ccl28* R	GCTCTCTGTGTCAATCCCAATA<br>CCGGTGTTTCTTTCTTCCCT	*ifn7* F<br>*ifn7* R	ACCTGTCGAGGTTGCATATTAG<br>GCAATGAGGGTGCAGAATTAG
cd4 F<br>*cd4* R	GTCGCCCTCCACTAATCTAATC<br>ACCGTTATCCCACGTTTATCC	*ifnl3* F<br>*ifnl3* R	GTCCTTTCAGCGATGGGATAA<br>ACGGCTTATGGCGAAACA
cd8 F<br>*cd8* R	GGCATTCACATTCACCCTAAAC<br>TAGCCCGGGACTGATAGAAA	*il10* F<br>*il10* R	CAGTCCGTGTCTGAAACAATTC<br>CAGCAACTTGTCCTTGAGAAAG
csf1r F<br>*csf1r* R	GAGCAAGGGCACTGATAGTT<br>AAAGGTCTACGGGCAAGATG	*il2ra* F<br>*il2ra* R	CAGCAGCCTTTCGTTGGAAC<br>TATTTCACAAGATTCTGAGTGCT
csf3r F<br>*csf3r* R	TGGATGAAGGACTACAGCTAATG<br>GCCTGTCATCTGTGAGGTTTA	*mcp* F<br>*mcp* R	GCCAAGACCGAGGACAC<br>GGACAAACCAACAGTAGAAACG
cd4 F<br>*cd4* R	GTCGCCCTCCACTAATCTAATC<br>ACCGTTATCCCACGTTTATCC	*rad2* F<br>*rad2* R	CATGTCAACGTCAAAGTCCAAG<br>TGTGCACCCTCTGGTTAAAG
cxcl8a F<br>*cxcl8a* R	CATAAAGACAGAAAGCAAGCCT<br>GGCTCCAAGCAGATATCG	*tgfb* F<br>*tgfb* R	CCTTACATCTGGAGCACAGATAC<br>GGAACACAGCAGGGAGAAAT
cxcl8b F<br>*cxcl8b* R	ACGACCCACTCTGCTTTC<br>TTTCTACCCAGCGCTGAG	*tnfa* F<br>*tnfa* R	TGTCAGGCAGGAAAGAAGCA<br>CAGCAGAGCAAAGAGGATGGT
cxcl10 F<br>*cxcl10* R	GGCTGTGATGAAGTTGAAGTTG<br>GTAGGGCAGTCATGAGTTTGT		

\
